# Three-Point Bending Behavior of Aluminum Foam Sandwich with Different Interface Bonding Methods

**DOI:** 10.3390/ma15196931

**Published:** 2022-10-06

**Authors:** Peng Huang, Xi Sun, Xixi Su, Qiang Gao, Zhanhao Feng, Guoyin Zu

**Affiliations:** School of Materials Science and Engineering, Northeastern University, Shenyang 110819, China

**Keywords:** aluminum foam, powder metallurgy, three-point bending, energy absorption

## Abstract

The interface bonding method has a great influence on the mechanical properties of aluminum foam sandwich (AFS). This study aims to investigate the effect of different interface bonding methods on the mechanical properties of AFS. In this paper, the metallurgical-bonding interface-formation mechanism of AFS prepared by powder metallurgy was investigated. The shear properties of metallurgical-bonded AFS were determined by the panel peeling test. The flexural properties and energy absorption of metallurgical-bonded and glued AFS were analyzed through the three-point bending test. The results show that the magnesium, silicon, and copper elements of the core layer diffuse to panels and form a metallurgical composite layer. The metallurgical-bonding strength between the panel and core layer is higher than that of the foam core layer. The peak load of metallurgically-bonded AFS is 24% more than that of glued AFS, and energy absorption is 12.2 times higher than that of glued AFS.

## 1. Introduction

Aluminum foam has many outstanding physicochemical properties, such as low density, high specific strength, high specific energy absorption capacity, sound absorption, and electromagnetic shielding [1,2,3,4,5]. Aluminum foam sandwich panels (AFS) are aluminum panels added on both sides of aluminum foam [3]. The aluminum panels on both sides can substantially improve the mechanical properties of the aluminum foam and expand its application scenarios. Therefore, AFS has a wide range of applications in construction, vehicles, ships, aerospace, and defense [6,7,8,9].

The bonding methods of the panel and core layer of AFS mainly include glued bonding and metallurgical bonding. Previously, there were more studies on glued AFS. Bart-Smith et al. [10] and McCormack et al. [11] conducted quasi-static three-point bending tests on glued AFS and found four failure modes: face yielding, core shear, indentation, and face wrinkling, and constructed the collapse mechanism maps. Crupi et al. [12] conducted three-point bending experiments on extruded composite AFS and glued AFS and found that the interface bonding method and span would affect the failure mode of the sandwich panel. Yu et al. [13] and Jiang et al. [14] investigated the effect of different panel thicknesses and core thicknesses on the failure modes of AFS and modified the quasi-static model. Tagarielli et al. [15,16] performed three-point bending experiments on AFS under simple support and clamped ends. The failure modes in the two cases were compared, and it was found that the ductility of the panels affects the ultimate strength of AFS. Zhang et al. [17,18] and Wang et al. [19] conducted three-point bending experiments on asymmetric AFS and found multiple failure modes of core shear. Pandey et al. [20] compared foam core layer and carbon fiber as panels of AFS and found that adding two sides of carbon fiber panel increased flexural load-bearing capacity by eight times and energy absorption by 58%. Sun et al. [21] also used carbon fibers as panels and inserted different aramid staple fibers into the face-core interface to investigate the failure modes and energy-absorption efficiency of AFS at different glued interfaces. Wang et al. [22] added glass fibers between the aluminum panel and the foam core layer and found that the addition of glass fibers improved the material′s overall performance. Wang et al. [23] used carbon fiber-reinforced plastic as panels and the effects of core layer thickness and density on the mechanical properties of AFS were investigated. Yan et al. [24] conducted three-point bending experiments on AFS prepared with different binders, and the results showed that the decrease in bond strength changed the failure mechanism of AFS and reduced their overall performance. The interfacial bond strength primarily affected the mechanical properties of AFS, and low interfacial strength resulted in debonding of the core layer from the panel [12,13,19,21,22,23,24], wrinkling of the panel [11,17], and short stress plateaus [17,21,22,24].

Some properties of the adhesives can affect the use scenario of glued AFS. Li et al. [25] conducted quasi-static three-point bending tests on AFS at different temperatures and found that the increase in temperature decreased the strength of the panels and core layers. The adhesive′s intolerance to high temperature also affected the mechanical properties of the sandwich panel. Pantelakis et al. [26] showed that disadvantages such as environmental aging, precontamination of bonded surfaces, poor durability, poor heat resistance, and moisture absorption limited the use of glued aluminum foam sandwich panels in aerospace applications.

The common methods for preparing metallurgical-bonded AFS are extrusion lamination [12], rolling lamination [27], and welding [28,29]. Wan et al. [28] used Zn alloy as a welding alloy with ultrasonic vibration to achieve metallurgical bonding of aluminum panels to core foam. The mechanical properties of the welded AFS are significantly improved compared to the simple aluminum foam. Ubertalli et al. [29] used Zn and Zn alloys as welding alloys to prepare AFS and compared the two samples for three-point bending experiments. The foam-aluminum sandwich panels produced from Zn alloys as welding materials were found to have higher stiffness. However, AFS prepared by the welding method has some limitations in terms of size, which is not conducive to actual industrial production.

Previous studies comparing the performance of metallurgical-bonded AFS and glued AFS are relatively few. Based on the study of Zu et al. [27], the metal-powder ratio, shell design, and rolling process [30] were optimized, and large-size metallurgical-bonded AFS were successfully prepared. In this paper, the metallurgical-bonding interface of AFS is characterized and analyzed, the interface peeling experiment of AFS is conducted, and the three-point bending performance of AFS under different interface bonding methods is compared.

## 2. Experimental Procedures

### 2.1. Materials and Precursor Preparation

In this study, the aluminum foam sandwich panel was prepared by the powder-metallurgy rolling method, and various powder ratios and particle sizes are shown in Table 1. TiH_2_ powder was used as the foaming agent in this experiment, and TiH_2_ was pre-treated in a muffle furnace at 470 °C for 1.5 h to allow TiH_2_ to reach its peak hydrogen release at a higher temperature. The upper and lower panels’ material is 3003 aluminum alloy plate. The size of the plate is 500 × 350 × 4 mm.

**Figure 1:**
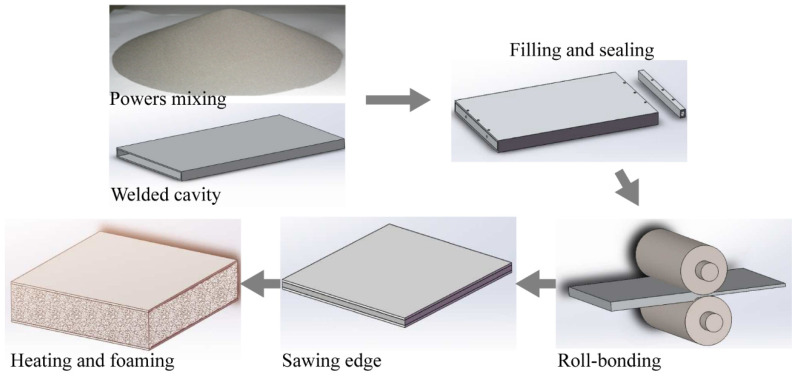
Process flow chart for preparation of AFS.

In this study, 5 mm steel mixing balls were used, and the mixing balls and powders (Al, Mg, Si, Cu, TiH_2_) were put into the SYH-600 mixer at a mass ratio of 1:1, with a mixing time of 6 h and a mixing speed of 8 r/min. Firstly, the aluminum alloy panel was soaked in aqueous sodium hydroxide solution with a concentration of 40 g/L for 10 min and then cleaned with dilute hydrochloric acid while removing the surface adhesion with a wire brush. The aluminum panel was welded with two 30 mm wide aluminum strips in the length direction and sealed with rivets at one end in the width direction. The well-mixed powder was loaded from the other end and sealed with rivets (Figure 1). The prepared aluminum alloy box size was 500 × 350 × 38 mm, and the thickness of the middle cavity was 20 mm.

Firstly, the aluminum alloy boxes loaded with powder were rolled at room temperature to discharge the gas in the powder and enhance the powder densities (Figure 1). The reduction of each pass of cold rolling was 1 mm, and the rolling rate was 6 m/min. The thickness of the box after cold rolling was about 24 mm; the thickness of the aluminum alloy panel was unchanged, and the core layer powder density was about 85%. Secondly, the boxes were hot rolled at 400 °C with 5 passes and a 20–30% pressing rate per pass to obtain precursors with a thickness of 6.5 mm. The precursors were held at 400 °C for 2 h to eliminate residual stresses. The precursors were cut into 200 × 200 × 6.5 mm size squares by wire cutting, and were put into steel molds with a height of 26 mm and foamed in a furnace at 620 °C for about 15 min. The edges of the AFS were excised by wire cutting because the edge structure was not uniform. Samples of size 170 × 50 × 26 mm were obtained for a three-point bending test.

The two panels and core layer of AFS by separated by wire cutting, then apply epoxy resin was glued evenly on the surface of the panels and the core layer, and this was let to stand for 12 h under 10 kg pressure, so that the panel and core layer could realize the glued bonding. By adjusting the height of the steel mold to 15 mm, AFS with a thickness of 15 mm could be obtained with the same foaming process. This thinner AFS was used for the interface peeling experiment.

### 2.2. Panel Peeling Test

**Figure 2:**
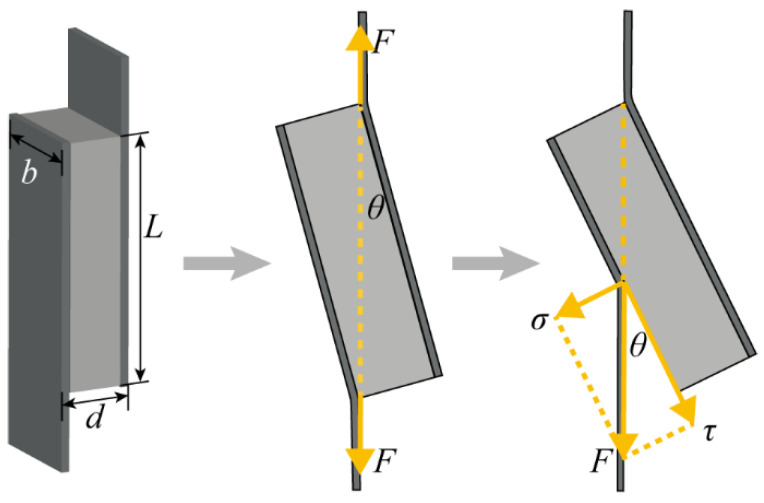
Diagram of interface peeling test.

To test the bonding strength of metallurgical-bonded AFS, we performed the panel peeling test. The panel peeling test of AFS was conducted at room temperature using the electronic universal testing machine AG-XPLUS (using standard ASTM C273-16). The schematic diagram of the panel peeling test is shown in Figure 2, where the fixture clamps both sides of the panels of metallurgical bonded AFS and applies vertical tension. As the tension increases, the sample will be deflected by a certain angle *θ*. The vertical tension *F* has a tangential stress *τ* along the panel direction. In this study, a thinner AFS was used to make the deflection angle *θ* as small as possible so that the tensile force would act on the interface as much as possible. The dimensions of AFS were 60 × 20 × 15 mm. At least three samples of metallurgical bonded AFS were used for this test. According to standard ASTM C273-16, the equation for the shear stress *τ* is:

(1) τ=F/(L×b)

where *F* is the tensile force; *L* is the length of the sample; *b* is the width of the sample.

### 2.3. Three-Point Bending Test

**Figure 3:**
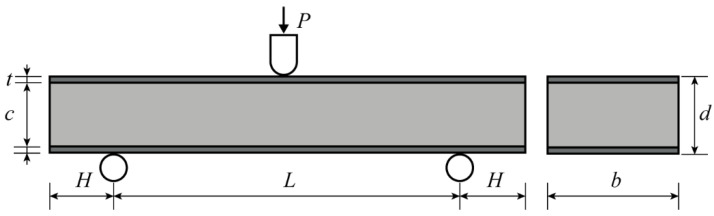
Sketch of AFS subjected to the three-point bending test.

The electronic universal testing machine AG-XPLUS was used to perform a three-point bending test on AFS at room temperature (using standard ASTM C393-06). The schematic diagram of the three-point bending test is shown in Figure 3. A cylindrical hammerhead (with a radius of 5 mm) applied a load *P* to the middle of the sample. The span between the two support points was *L*, and the distance between the two ends of the sandwich panel beyond the support points was *H*. The thickness and width of the sandwich panel, the thickness of the panel, and the thickness of the core layer were *d*, *b*, *t*, and *c*. The values of each parameter are shown in Table 2. The core layer thickness of glued AFS was thicker than that of metallurgical bonded AFS, and the total weight of glued AFS was more significant because of the epoxy resin adhesive used in glued AFS. The density of the core layer was obtained by subtracting the weight of the panel from the total mass of AFS and dividing it by the volume of the core layer, where the density of the aluminum panel was taken as 2.73 g/cm^3^. The hammerhead depression rate was 3 mm/min in the three-point bending test. A digital camera was used to take a photo every 10 s during the experiment to record the deformation process of the samples. Metallurgical-bonded and glued AFS were each subjected to 3 sets of experiments.

## 3. Results and Discussion

### 3.1. Microstructure of Metallurgical Composite Interfaces

**Figure 4:**
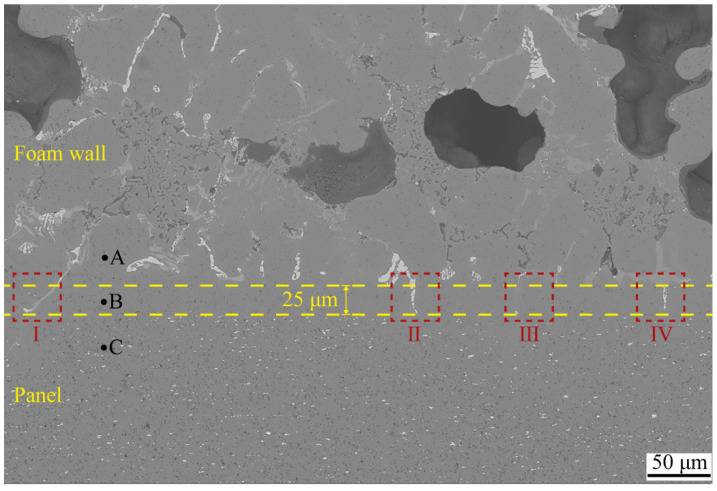
SEM micrograph of the core–panel interface.

**Figure 5:**
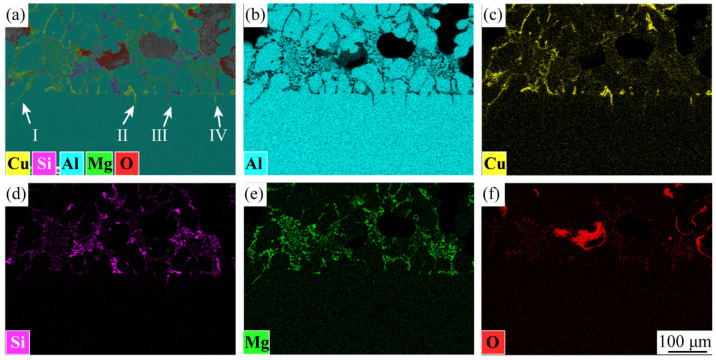
Distribution of elements at the core–panel interface: (**a**) all elements distribution, (**b**) Al distribution, (**c**) Cu distribution, (**d**) Si distribution, (**e**) Mg distribution, (**f**) O distribution.

**Figure 6:**
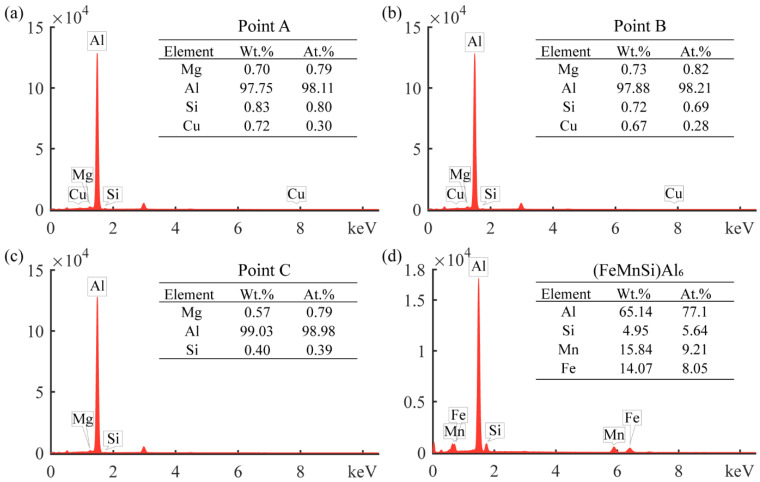
EDS results in different areas: (**a**) cell wall, (**b**) metallurgical composite layer, (**c**) panel, (**d**) white phase in the panel.

The interfacial structure of the aluminum foam core layer and the panel played a significant influence on the mechanical properties. The SEM morphology of the interface of metallurgical-bonded AFS in this experiment is shown in Figure 4, and the distribution of each element at the interface is shown in Figure 5. The aluminum panel was made of 3-series aluminum alloy. From Figure 4, it can be observed that there was a uniformly distributed white phase in the panel with a size of about 2–3 μm, which was identified as the (FeMnSi) Al_6_ phase (composition as shown in Figure 6d) by energy spectrum analysis. A variety of alloy phases were formed at the bubble wall, and it could be observed from Figure 4 that the alloy phases were distributed along a straight line at the interface, and it could also be observed from Figure 5 that there were apparent boundaries in the distribution of Cu, Si, and Mg elements. A metallurgical composite layer of about 25 μm existed between the panel and the bubble wall, bounded by the alloy phase and the (FeMnSi) Al_6_ phase (Figure 4). The cell wall (Point A), metallurgical composite layer (Point B), and panel (Point C) were analyzed by energy spectrum to determine their elemental types and contents (Figure 6). The results showed that the metallurgical composite layer contained the same metal elements as the bubble wall, and the Si, Cu, and Mg elements in the core layer diffuse to the panel.

**Figure 7:**
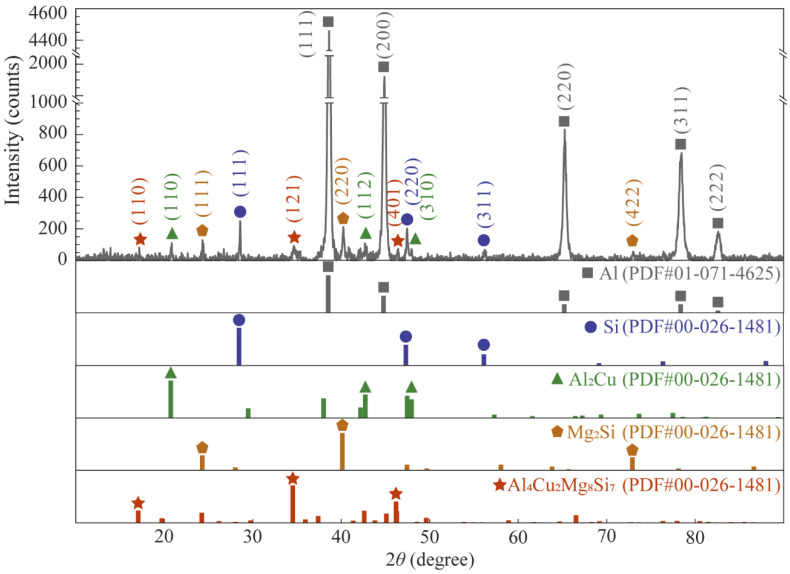
XRD pattern of the core layer foam.

As shown in Figure 4, there were long strips of alloy phases (Ⅰ Ⅱ Ⅲ Ⅳ) in the metallurgical composite layer, and these alloy phases were also present in the cell walls. The foam core layer was ground into a 300 mesh powder with a ceramic mortar and subjected to XRD physical-phase examination to determine the alloy phase composition in the cell walls (Figure 7). The results show that the main phases were Al monomer, Si monomer, Al_2_Cu, Mg_2_Si, and Al_4_Cu_2_Mg_8_Si_7_.

**Figure 8:**
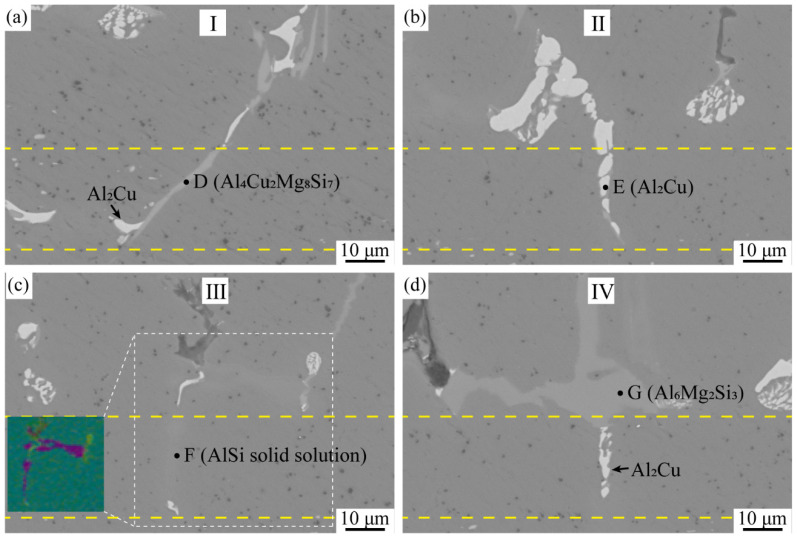
SEM micrograph of the metallurgical composite layer: (**a**) area I, (**b**) area II, (**c**) area III, (**d**) area IV.

**Figure 9:**
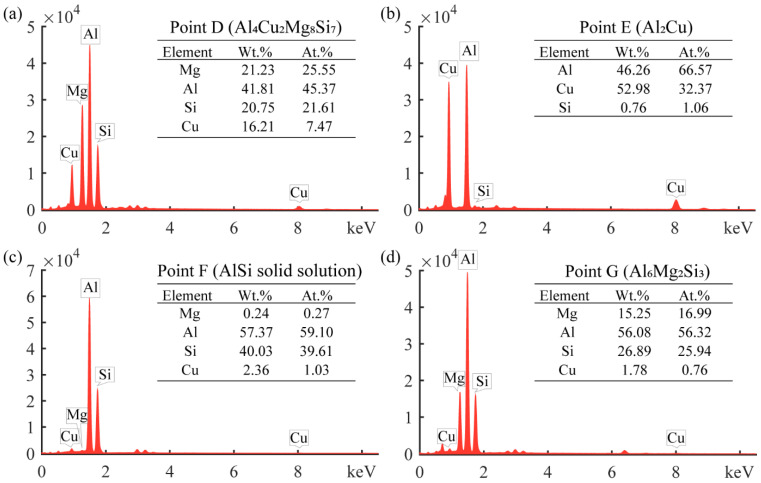
EDS results of the metallurgical composite layer: (**a**) point D, (**b**) point E, (**c**) point F, (**d**) point G.

The alloy phase morphology in the metallurgical-composite layer is shown in Figure 8, and the elemental composition of each alloy phase is shown in Figure 9. Combined with EDS and XRD analysis, the long gray alloy phase in Figure 8a can be identified as Al_4_Cu_2_Mg_8_Si_7_, and the small amount of white phase is Al_2_Cu. The white alloy phase in Figure 8b is the Al_2_Cu phase, which is present as a block at the cell walls, and the Al_2_Cu phase is in long strips at the metallurgical composite layer, indicating that the Cu element diffuses toward the panel at the cell wall. The alloy phase in Figure 8c is the solid solution phase of Al and Si, which is the diffusion of Si monomers at the interface to the panel during the foaming process and, finally, the formation of the AlSi solid solution phase at the metallurgical composite layer. The gray phase at the cell walls in Figure 8d is the alloy phase of AlSiMg, whose composition approximates Al_6_Mg_2_Si_3_, and the phase in the metallurgical composite layer is Al_2_Cu. The reason for the formation of this phase is that the Cu powder, Mg powder, and Si powder in the precast billet gathered at the interface, but the Cu powder was close to the panel, and during the foaming process, the Cu elements diffused toward the panel and grew into the Al_2_Cu phase, and the Cu, Mg and Si elements at the cell wall formed the Al_6_Mg_2_Si_3_ alloy phase. The alloy phases of the metallurgical composite layers were all elongated due to the diffusion of these added metal elements in the direction of the panels during the foaming process, resulting in the formation of the elongated alloy phases. From the alloy phase and element distribution at the interface, the experimentally prepared AFS achieves interfacial metallurgical bonding, and the bonding interface is relatively flat without defects such as voids.

### 3.2. Panel Peeling Test

**Figure 10:**
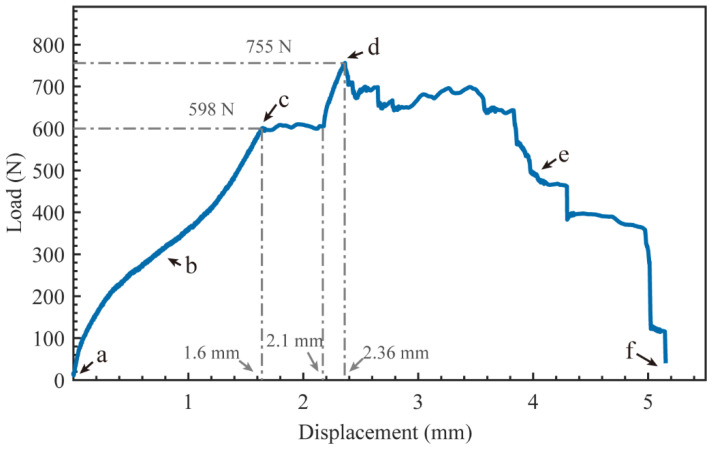
Load-displacement curves of metallurgical bonded AFS at the panel peeling test.

**Figure 11:**
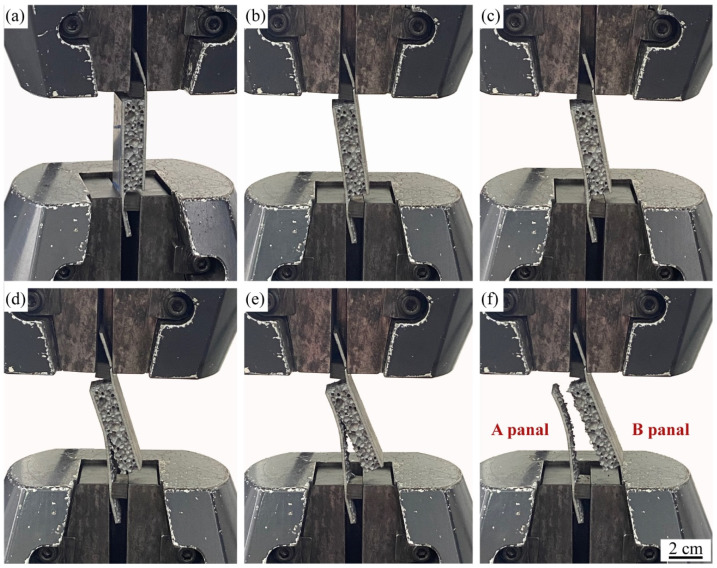
Series images of the panel peeling teat of metallurgical bonded AFS: (**a**) 0 mm, (**b**) 0.8 mm, (**c**) 1.6 mm, (**d**) 2.4 mm, (**e**) 4.0 mm, (**f**) 5.1 mm.

**Figure 12:**
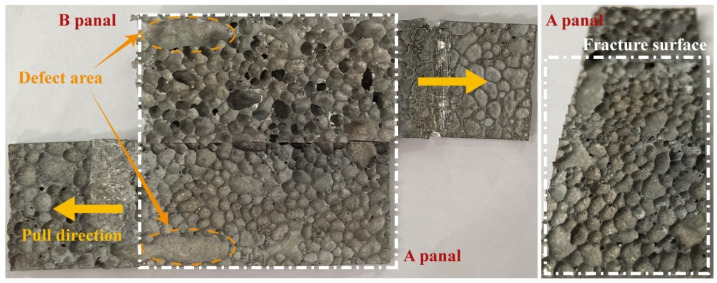
Macroscopic morphology of fracture area of metallurgical bonded AFS after stretching.

The panel peeling tests were performed on the samples to test the metallurgical-bonding strength of the panel and core layer of AFS. The load-displacement curve of a representative sample in the panel peeling test is shown in Figure 10, and the deformation failure behavior of the sample is shown in Figure 11. The individual states a–f in Figure 10 correspond to Figure 11a–f. The individual states a–f in Figure 10 correspond to Figure 11a–f. Figure 11a shows the initial state of the sample, and as the stretching proceeds, the sample is deflected by a certain angle *θ* (Figure 11b). The panel separation from the core layer starts in Figure 11c when the sample is rotated at an angle of 14 degrees and the load is 598 N. Then, a stress plateau appears with tensile displacements between 1.6 mm and 2.1 mm. The reason for this plateau is that the aluminum foam sandwich panel has a large bubble hole-type defect area at this location (such as the yellow dashed area in Figure 12), which leads to a decrease in the interfacial bonding strength between the core and the panel. The load in Figure 11d reaches a maximum value at which the load is 755 N. As the tensile deformation increases, the tensile stress decreases, and the sample rotation angle *θ* increases until it is completely disconnected, as shown in Figure 11f.

The macroscopic morphology of the fracture surfaces of panels A and B in Figure 11f is shown in Figure 12, and it can be found that the fracture location is not at the composite interface, and the fractured cell walls are attached to the panels after the fracture. The tensile failure mode of AFS is the shear failure of the core layer. According to Equation 1, the maximum shear strength that the foam core layer can withstand can be calculated as 0.629 MPa. The tensile test results show that the bonding interface strength between the panel and the core layer is higher than the strength of the core layer, and the interface achieves the ideal metallurgical bonding.

### 3.3. Three-Point Bending Test

**Figure 13:**
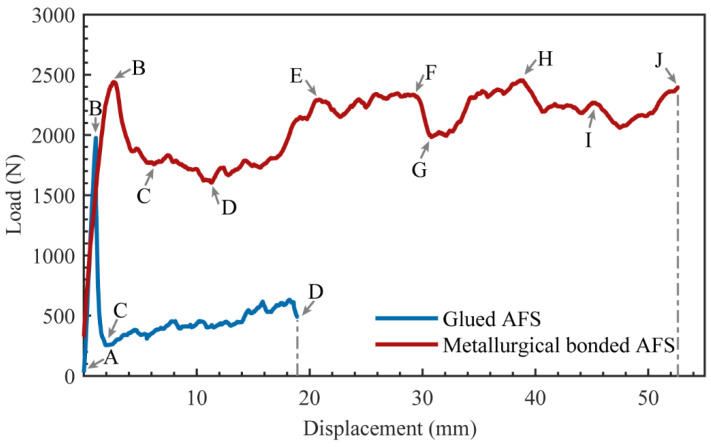
Load-displacement curves of metallurgical bonded and glued AFS in the three-point bending experiment.

**Figure 14:**
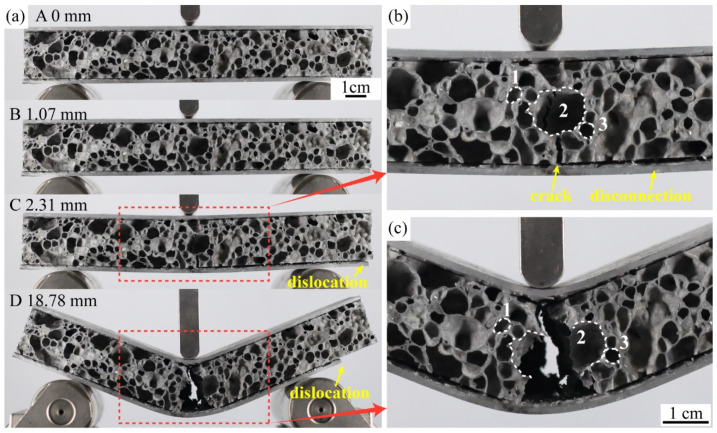
**(a)** The sequence of images shows the three-point bending test of glued AFS, (**b**) displacement is 2.31 mm, (**c**) displacement is 18.78 mm.

The load-displacement curves of a representative metallurgical-bonded sample and a glued sample in the three-point bending tests are shown in Figure 13. The points A to D in the load-displacement curve of the glued AFS correspond to A to D in Figure 14a. During the process from point A to point B, the glued AFS is deformed elastically and then deformed plastically, and the load reaches the maximum value of 1975.6 N at point B. The displacement at this time is 1.07 mm. The deformation of the sandwich plate at point B is not apparent (Figure 14a). The load drops sharply during the process from point B to point C. As shown in Figure 14a,b, a crack in the middle of the core layer of AFS in the vertical direction appeared at point C. The core layer and the lower panel appeared to be debonded, and the right end of the sandwich panel appeared to be dislocated from the panel. From point C to point D, the crack in the middle of the core layer expands, and the dislocation between the core layer and the lower panel increases. As shown in Figure 14b,c, cell 2 in the middle of the core layer ruptured during this process, although cells 1 and 3 did not undergo deformation. Except for the middle cells of the core layer, the remaining cells did not play a role in energy absorption. The same failure mode as in this test had been observed in several studies [13,21,22,24] that investigated glued AFS. Therefore, the interfacial bonding strength of glued AFS affects the failure mode of sandwich panels.

**Figure 15:**
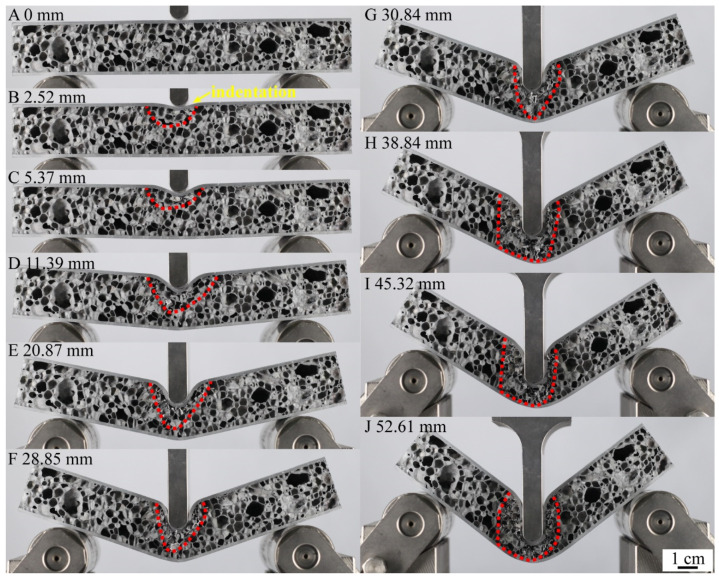
The sequence of images showing the three-point bending experiment of metallurgical bonding AFS.

**Figure 16:**
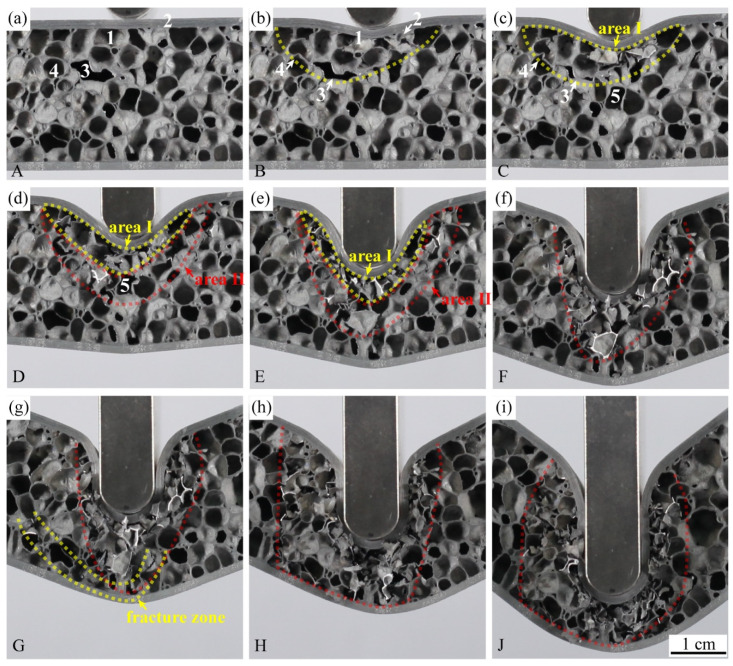
Deformation state of AFS under different compression amounts: (**a**) 0 mm, (**b**) 2.52 mm, (**c**) 5.37 mm, (**d**) 11.39 mm, (**e**) 20.87 mm, (**f**) 28.85 mm, (**g**) 30.84 mm, (**h**) 38.84 mm, (**i**) 52.61 mm.

The points A-J of the load-displacement curve of metallurgical-bonded AFS in Figure 13 corresponded to the points A-J in Figure 15 and Figure 16. The deformation process of AFS in the three-point bending test is shown in Figure 14. The deformation area of the core layer extends from the upper half to the whole middle, and only the middle core layer was deformed during the whole deformation process, and the core layers on both sides were not deformed. Point A to point B was deformed elastically and then deformed plastically, and point B reached a maximum load of 2423.3 N and a displacement of 2.52 mm. The upper part of the core foam was deformed during this process, and the lower part was not deformed. As shown in Figure 16b, cell 1 near the upper panel shows deformation, and hole 2 shows a cell wall fracture. Cell 3, away from the upper panel, shows compression deformation in the vertical direction, and cell 4 shows a bending of the bubble wall. The metallurgical-bonded AFS had a larger displacement when it reaches its highest value than the glued AFS failure process. This was due to the indentation of the upper panel of the metallurgical bonded AFS at point B.

The load decreased during the process of point B to point C. As shown in Figure 16c, cells 1 and 2 near the middle of the upper panel were completely ruptured, cell 3 away from the upper panel was further compressed, and the cell wall of cell 4 was fractured. Most of the cells in the deformation area Ⅰ ruptured, reducing the ability of AFS to withstand the load. During this process, the upper panel was indented, and the upper part of the core layer was deformed, but the lower part of the core layer and the lower panel were not deformed. This deformation process was different from the deformation process of glued AFS. Glued AFS, due to the low bonding strength of the panel and core layer, there was a dislocation of the panel and core layer when compressed, the core layer foam was broken from the middle, and the core layer bubble hole was not deformed, so that the upper and lower panels of glued AFS were bent and deformed at the same time.

The load decreased slowly from point C to point D. All the cells in the deformation region I were compressed and ruptured at point D, as shown in Figure 16c,d. The ability of the core layer in the deformation region I to withstand the load was further reduced. At the same time, a new deformation region II appeared below the deformation region Ⅰ. For example, cell 5 was compressed and deformed. The appearance of deformation region II slowed down the decreasing trend of the load.

The load rose from point D to point E because the ruptured vesicles in the deformation region I were compacted (Figure 16e). The core density rose, and the capacity to carry the load rose. The deformation region II expanded further to the middle and lower part of the core layer. The load remained relatively stable during the process from point E to point F, and a stress plateau appeared. The whole deformation area did not become larger during this process. The cells in the deformation region were further compacted when the vesicle rupture occurred, leaving the core layer′s ability to withstand the load in a relatively balanced state.

The load dropped during the process from point F to point G. This was because a fracture zone appeared in the core layer near the lower panel at point G (Figure 16g). At this point, the entire central core layer of aluminum foam was deformed. The generation of the core fracture zone made the core layer less capable of withstanding the load. As the hammerhead continued to press down, the fracture zone extended to the upper panel, causing the deformation region to expand further (Figure 16h). Point H to point J was the process of the deformation area bubble cells being compressed, ruptured, and compacted, and the load was always at a high level.

During the three-point bending test, the middle core layer of the metallurgical bonded sample was gradually deformed in the sub-region, which played a good role in buffering energy absorption.

### 3.4. Bending Stiffness and Energy Absorption

The bending stiffness (R) of metallurgical bonded and glued AFS was calculated using the following equation [23].

(2) $$R=\frac{P_{max}L^{3}}{48d_{max}}$$

where *P_max_* is the peak load carried by the test sample before failure, *L* is the span length and *d_max_* is the displacement at the peak load. The peak load of the metallurgical-bonded AFS wa2432.3 N, and the displacement was 2.52 mm (as shown in Figure 13). The peak load of the glued AFS was 1975.6 N, and the displacement was 1.07 mm. The peak load of the metallurgical bonded AFS was 18.8% higher than that of the glued AFS. The bending stiffnesses of the metallurgical bonded and glued AFS were calculated to be 34.75 N·m^2^ and 66.47 N·m^2^, respectively, according to Equation 2. The bending stiffness of glued AFS was higher because the upper panel deformed less when the sample failure occurred, and the addition of epoxy resin glue increased the density of the core layer.

The area of the load-displacement curve was calculated to find the energy absorption *E* when the sample was subjected to a three-point bending test, and the specific energy absorption *E_s_* was obtained by dividing *E* by the sample mass [31].

(3) $$E=\int_{0}^{d}Fds$$

(4) $$E_{s}=E/m$$

where *E* was the energy absorption of the sample when the deformation was *d*; *F* was the bending force; *m* was the mass of the sample.

**Figure 17:**
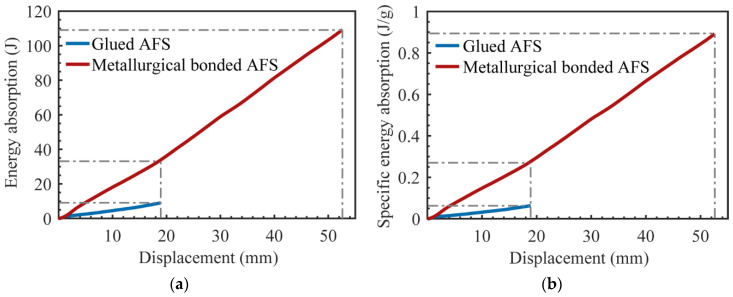
Energy absorption (**a**) and the specific energy absorption (**b**) of AFS.

The glued samples used epoxy resin glue, which increased the weight of the samples. The average mass of the metallurgical bonded AFS was 122.5 g, and the average mass of the glued AFS was 142.4 g. Energy absorption curves and the specific energy absorption curves of AFS are shown in Figure 17. When the deformation was 18.8 mm, the energy absorption of metallurgical bonded AFS was 33.6 J, and the energy absorption of glued aluminum foam sandwich panel was 8.95 J. The energy efficiency of the metallurgical bonded AFS was 3.75 times that of the glued AFS. Moreover, the metallurgical bonded AFS had a more extended stress plateau and could withstand higher loads until the deflection was 52.6 mm with a load of 2394.2 N, which was only 2.4% lower than the peak load of 2453.2 N. The energy absorption of the metallurgical bonded AFS during the whole deformation process was 109.3 J, which was 12.2 times the energy absorption of the glued AFS.

When the deformation was 18.8 mm, the specific energy absorption of the metallurgical-bonded AFS was 0.274 J/g, and that of the glued AFS is 0.0628 J/g. The specific energy absorption efficiency of the metallurgical-bonded AFS was 4.36 times that of the glued AFS. Throughout the deformation process, the specific energy absorption of the metallurgical-bonded AFS is 0.892 J/g, which was 14.2 times higher than that of the glued AFS. The experimental results show that the energy absorption capacity of metallurgical-bonded AFS was significantly stronger than that of glued AFS.

## 4. Conclusions

Here in this paper, large-size aluminum foam sandwich panels with metallurgically-bonded interfaces were prepared by powder metallurgy to investigate the mechanism of interface formation. Panel peeling tests and three-point bending tests at quasi-static conditions were carried out to explore the effect of the interfacial bonding mode on the mechanical properties. The key conclusions can be summarized as follows:

1. A metallurgical composite layer of 25 μm formed at the junction of the panel and the core layer. Cu, Mg, and Si near the panel during the foaming process diffused toward the panel, forming Al_2_Cu, Mg_2_Si, and Al_4_Cu_2_Mg_8_Si_7_ alloy phases. All these alloy phases were present in the metallurgical composite layer in the form of long strips.
2. The panel peeling test was conducted on metallurgical-bonded AFS with a peak load of 755 N. The failure mode of the sample was tearing the core layer, indicating that the bonding strength of the panel and the core layer was higher than the strength of the core layer.
3. The glued AFS appeared to debond the core layer from the lower panel in the three-point bending test, and the core layer was fractured only in the middle, and the load-bearing capacity was greatly reduced. The metallurgically-bonded AFS gradually produced breakage and compaction of the core layer in the three-point bending test, which played a good role in bearing the load.
4. Metallurgical-bonded AFS had higher peak loads and plateau stresses in the three-point bending tests, and the stress plateaus lasted longer. During the whole deformation process, the energy absorption of the metallurgically-bonded AFS was 12.2 times higher than that of the glued AFS, and the specific energy absorption was 14.2 times higher. The energy absorption efficiency of metallurgical bonded AFS was much higher than that of glued AFS.

## Figures and Tables

**Table 1:** Elemental composition of mixed powders [30].

Composition	Range Size (μm)	Purity (%)	Content
Al	<45	99.7	85%
Si	<38	99.5	6%
Mg	<75	99.9	4%
Cu	<38	99.9	4%
TiH_2_	<45	99.7	1%

**Table 2:** Dimension of specimen geometry was considered during the three-point bending test.

Joining Method	*L* (mm)	*L* + 2*H* (mm)	*d* (mm)	*b* (mm)	*t* (mm)	*c* (mm)	Total Mass (g)	Core Density (g/cm^3^)
glued bonding	120	170	28.26	50	1.59	25.06	142.38	0.32
Metallurgical bonding	120	170	26.32	50	1.62	23.07	122.5	0.24
